# CAROTID SINUS SYNDROME IN A PATIENT WITH ADVANCED LARYNGEAL CANCER

**DOI:** 10.51894/001c.123087

**Published:** 2024-08-30

**Authors:** Montaser Elkholy

**Affiliations:** 1 Internal Medicine Detroit Medical Center Sinai-Grace Hospital

## 79

### INTRODUCTION

An important regulatory mechanism for maintaining arterial blood pressure in the body is the carotid sinus reflex. Parry observed in 1799 that placing pressure on one of the carotid sinuses can slow down the pulse rate. Carotid sinus hypersensitivity (CSH) is the term for an amplification of the response to this maneuver. Rarely, head and neck tumors can cause carotid sinus syndrome (CSS). We describe a case with advanced laryngeal carcinoma presented with intermittent episodes of bradycardia and hypotension secondary to CSS.

### CASE DESCRIPTION

A 68-year-old male with past medical history of Stage 4 laryngeal squamous carcinoma presented to the hospital after syncopal attack. On admission, his vitals showed a heart rate of 95/min and a blood pressure of 101/70 mmHg. On physical examination, there was a fixed and firm 5cm, as well as a palpable 5cm submandibular mass. Routine blood tests including electrolytes, glucose, liver, kidney, thyroid function, and high-sensitivity troponin T were within normal. EKG showed normal sinus rhythm. CT head showed no evidence of acute intracranial abnormality. Cerebral CT angiogram demonstrates normal contrast enhancement of the major intracranial arteries without occlusion, stenosis, or filling defects. CTA neck showed a large heterogeneously enhancing mass in the right lateral neck extending from the clavicle to the supraclavicular region measuring 6.6 x 5.5 x 8.4cm. The mass encases the right common and internal carotid arteries. During hospital course, the patient developed recurrent attacks of hypotension and bradycardia with full recovery in a few minutes. No specific triggers were apparent. No elevation of myocardial necrosis markers or electrolyte imbalance. Transthoracic echocardiography showed no abnormal findings. We noticed that during these attacks when we turned the patient’s neck to the left (opposite site of lesion), heart rate returned to normal. A diagnosis of CSS from tumor compression was assumed.

### DISCUSSION/CONCLUSION

Rare cases reported for carotid sinus syndrome in head and neck cancer patients. Our case is unique as patient developed mixed cardioinhibitory and vasodepressor subtype of CSS. Unexplained repeated episodes of bradycardia and hypotension in patients with head and neck cancer should notify the physician about the potential for carotid sinus compression.

